# Wide Resection and Iliac Crest Arthrodesis for Multiply Recurrent Giant Cell Tumor of First Metatarsal

**DOI:** 10.1155/2018/4521841

**Published:** 2018-12-03

**Authors:** Andrew Golz, Michael Murphy, Lukas Nystrom, Adam Schiff

**Affiliations:** ^1^Loyola University Medical Center, 2160 S 1st Ave. Maywood, IL 60153, USA; ^2^Cleveland Clinic, 9500 Euclid Ave. Cleveland, OH 44195, USA

## Abstract

**Case:**

Giant cell tumor of bone is a benign, aggressive neoplasm commonly arising in the femur, tibia, and distal radius and less commonly in the hands and feet. We describe a 22-year-old woman who underwent wide resection of multiply recurrent first metatarsal giant cell tumor and reconstruction with iliac crest arthrodesis.

**Conclusion:**

To our knowledge, there have been no previous reports of managing multiply recurrent giant cell tumor of the first metatarsal. The patient was without pain and exercising without difficulty 18 months following surgery. This method appears useful for reconstructing the foot following multiply recurrent giant cell tumor of the metatarsal.

## 1. Introduction

Giant cell tumor of bone (GCTB) is a benign, aggressive neoplasm with metastatic and high local recurrence potential. Histologically, these lesions consist of cytologically benign mononuclear round, ovoid, or spindle cells mixed with evenly distributed osteoclast-like multinucleated giant cells. GCTB makes up 20% of benign bone tumors and 5% of all primary bone tumors [1]. This condition most commonly occurs in patients 30–50 years old, with 90% of the cases arising in patients after the second decade of life and only 2–7.5% of the cohort in patients with open physes [2–4]. Some series show slight female predilection [1].

GCTB most commonly occurs in the long bones, namely the distal femur, proximal tibia, and distal radius, altogether accounting for 50% of the cases [2]. In the foot, the most commonly involved bone is the talus, followed by the calcaneus, with rare occurrence in the metatarsals [5]. Giant cell tumors (GCT) of small bones are reportedly more aggressive than GCT of long bones and can have high recurrence rates [6].

In terms of treatment, nonoperative methods are being used more frequently, including the use of denosumab, which is approved by the U.S. Food and Drug Administration for use in unresectable GCTB. However, operative treatment is most commonly pursued, and first-line treatment is intralesional curettage. Local adjuvants are often added to decrease the recurrence rate, and multiple studies suggest its increased efficacy compared to curettage alone [6–8]. En bloc excision and even amputation can be used for recurrent or recalcitrant cases. Regardless of treatment, the rate of recurrence of GCT of small bones trends toward the higher end of the ranges for all bones: 27–65% after curettage alone, 12–34% after curettage with adjuvants, and 0–12% after complete resection [2, 6, 8–11].

There are no detailed reports in the literature regarding the management of multiply recurrent giant cell tumor of the first metatarsal. We report a case of a second recurrence of giant cell tumor of the first metatarsal in a female who was treated successfully with wide resection and iliac crest arthrodesis. The following technique shows promise for addressing this difficult clinical problem.

## 2. Case Report

A 22-year-old woman with a known history of giant cell tumor of the left first metatarsal was evaluated for worsening left medial midfoot pain. She had previously undergone curettage and polymethylmethacrylate (PMMA) cementation of a biopsy-proven giant cell tumor of the first metatarsal two and a half years prior at a different institution. Six months following the index surgery, she had a recurrence treated with repeat curettage, liquid nitrogen local adjuvant, and placement of bone substitute graft. After the second procedure, she was ambulatory, but occasionally used a boot for comfort and avoided high-impact activities. Physical examination demonstrated a prominent first metatarsal base and a well-healed dorsal incision. She exhibited tenderness to palpation throughout the area of the scar.

**Figure 1:**
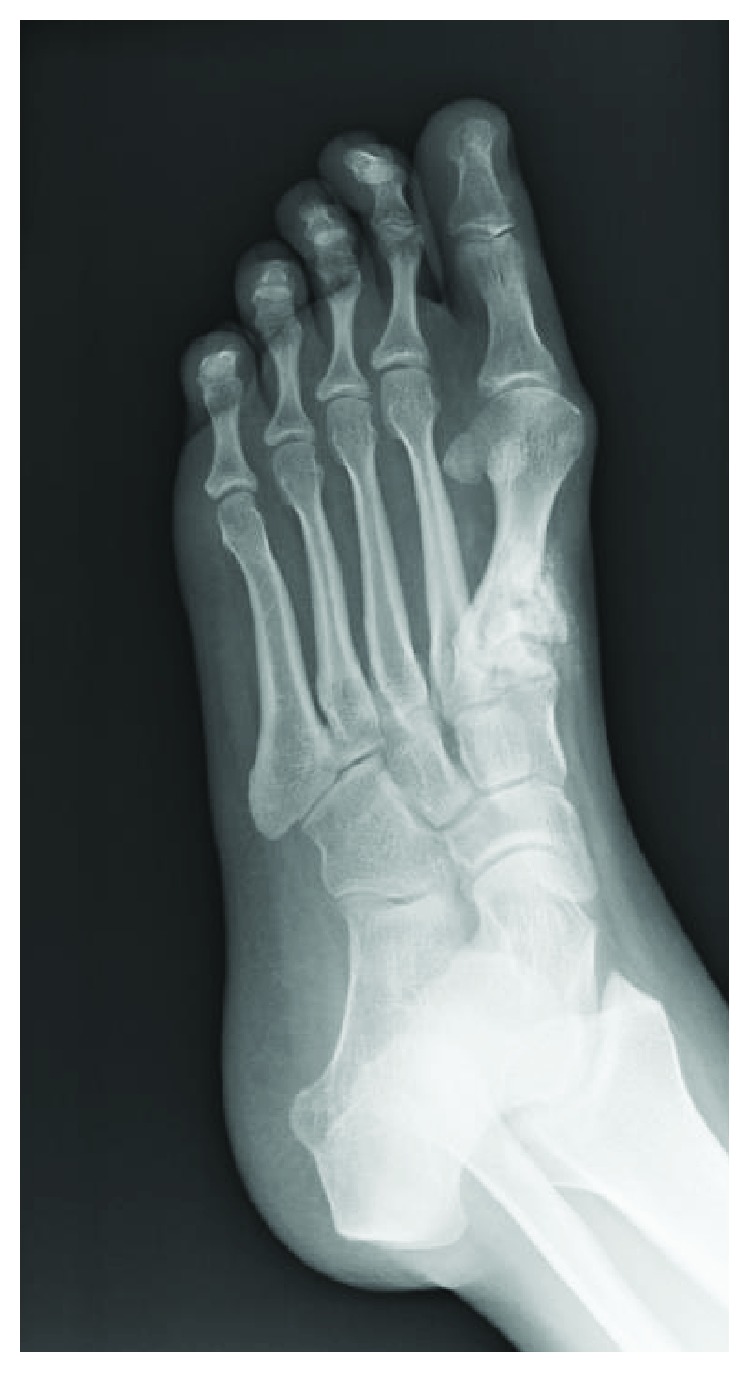
Plain radiographs of the left foot showing an expansile, radiolucent lesion of the first metatarsal base surrounding the known cement implantation of the first metatarsal.

**Figure 2:**
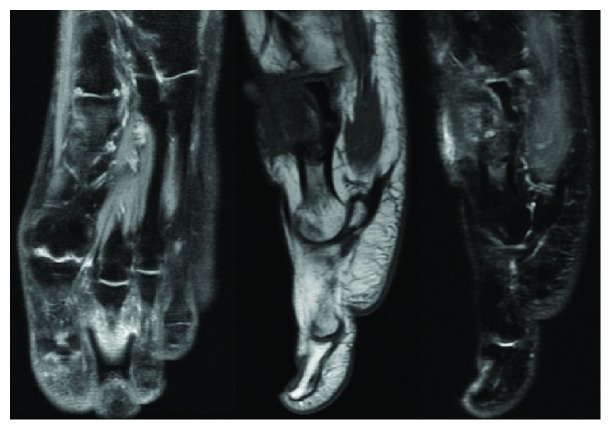
Magnetic resonance imaging showing a large soft-tissue mass surrounding the first metatarsal circumferentially and abutting the second metatarsal with high T2 signal and no T1 signal changes at the base of the second metatarsal.

**Figure 3:**
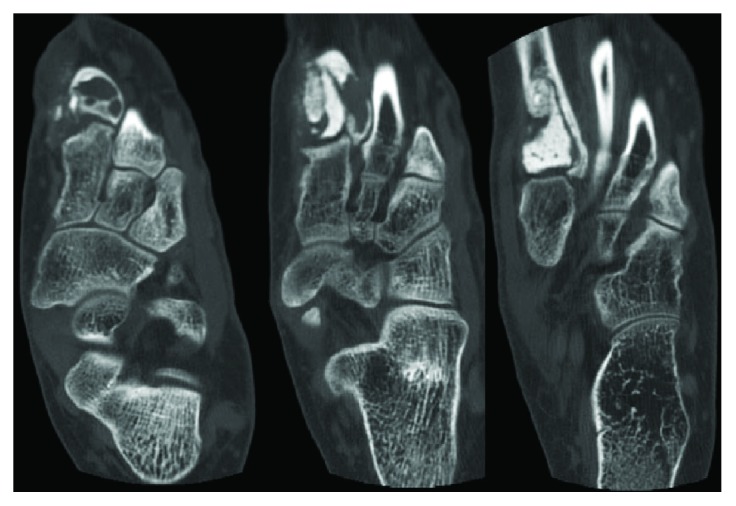
Computed tomography (CT) scan demonstrating an expansile lucency at the proximal aspect of the first metatarsal base toward the dorsal lateral side.

Radiographs of the left foot showed an expansile, radiolucent lesion of the first metatarsal base surrounding the known cement implantation of the first metatarsal (Figure 1). Magnetic resonance imaging showed a large soft-tissue mass surrounding the first metatarsal circumferentially and abutting the second metatarsal (Figure 2). There was high T2 signal with no T1 signal changes at the base of the second metatarsal. Computed tomography (CT) scan demonstrated an expansile lucency at the proximal aspect of the first metatarsal base toward the dorsal lateral side (Figure 3). There were some areas of cortical breach, and the bone was expanded and abutting the base of the second metatarsal. There was no evidence of direct extension into the second metatarsal or areas of osteolysis within the second metatarsal base. She was diagnosed with a second recurrence of giant cell tumor of bone of the first metatarsal base. As the tumor was recurrent, with destruction of the articular surface and little residual bone of the first metatarsal base, a wide resection of the tumor was recommended.

### 2.1. Surgical Technique

**Figure 4:**
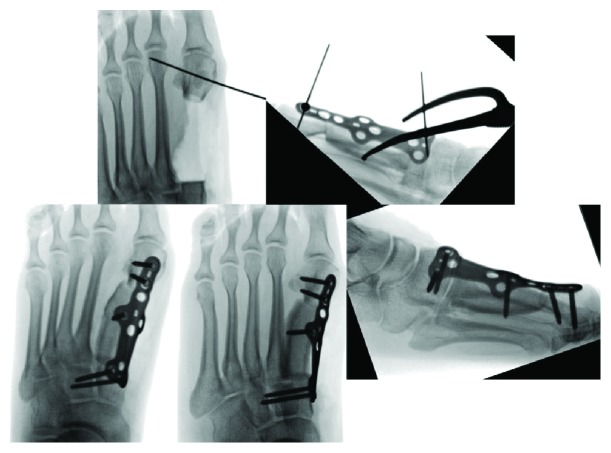
Intraoperative imaging demonstrating a wide resection disarticulating the TMT joint, placement of the graft in an appropriate position, and plate and screw fixation.

**Figure 5:**
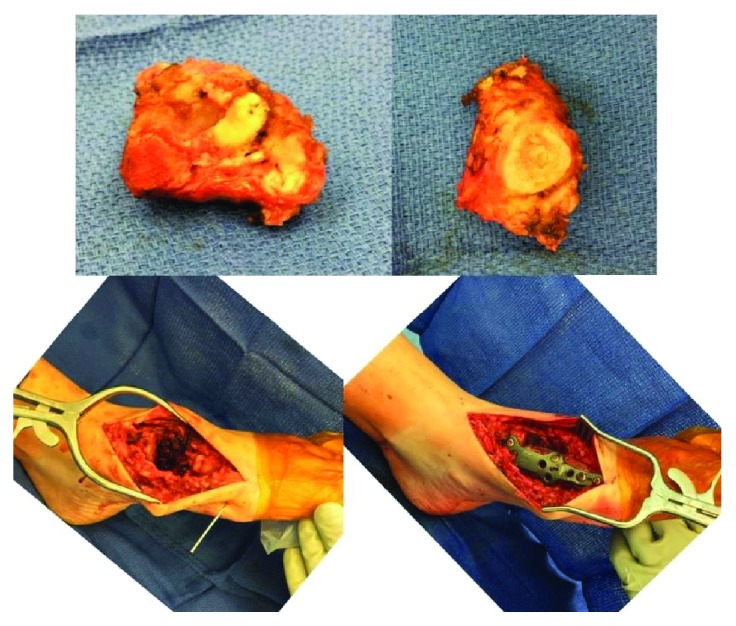
Intraoperative photographs demonstrating wide resection of proximal metatarsal and reconstruction with a TMT arthrodesis plate.

The procedure began by harvesting iliac crest bone graft (5 cm in length, 2 cm in depth). The prior dorsal incision was extended along the entire first metatarsal and proximally across the tarsometatarsal (TMT) joint. A wide resection was performed by disarticulating the TMT joint, maintaining periosteal coverage surrounding the metatarsal, and osteotomizing the metatarsal about 4.5 cm from the TMT joint. The damaged cuneiform articular surface (from articulation with cement) was resected with a wafer osteotomy. The resection bed was treated with adjuvant thermal necrosis. Margins were extended further with 10% hydrogen peroxide. The first toe was held in appropriate length and rotation and pinned to the second metatarsal. The iliac crest graft was fashioned to fit appropriately, contouring the angles for an appropriate junction. The graft was secured with an anatomic plate and screw construct (Figures 4 and 5).

### 2.2. Postoperative Course and Follow-Up

**Figure 6:**
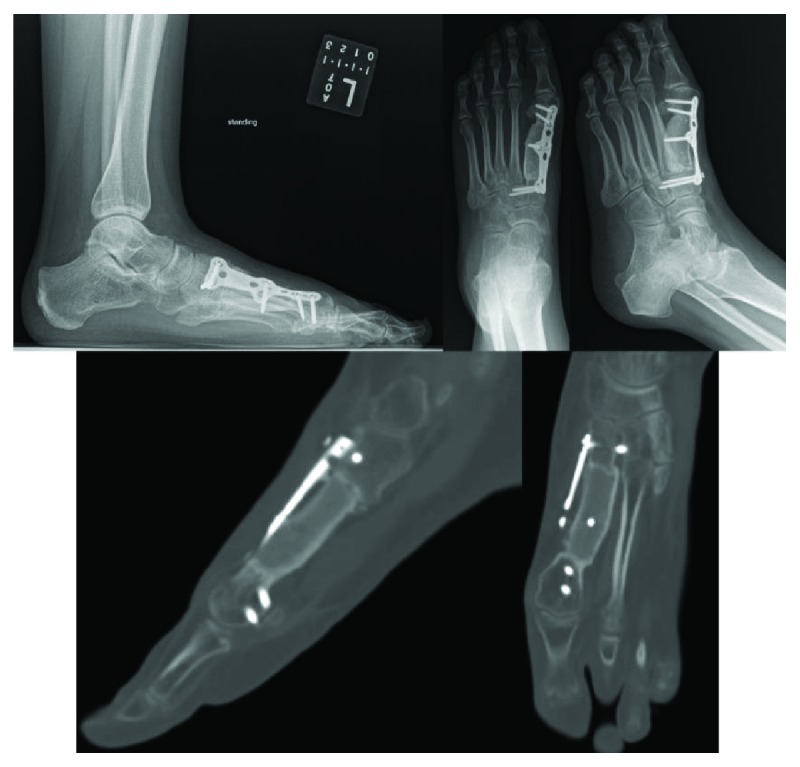
Nine-month postoperative plain radiographs and CT scan demonstrating osseous bridging at both the proximal and distal graft sites with no signs of tumor recurrence.

Nonweight bearing was maintained until the three month postoperative visit, at which time she began weight bearing as tolerated through the heel and 50% weight bearing through the forefoot with a removable boot. Four months following her operation, she was advanced to full weight bearing as tolerated in the boot. Five months after surgery, she began weight bearing as tolerated in a regular shoe and nonimpact exercises. Nine months after surgery, plain radiographs and CT demonstrated osseous bridging at both the proximal and distal graft sites with no signs of tumor recurrence (Figure 6). At one year following surgery, she denied pain in the left foot and was able to complete all activities of daily living without difficulty. Eighteen months following surgery, the patient had no left foot pain, was exercising without difficulty, and there was no radiographic evidence of recurrence. The patient was informed that data concerning the case would be submitted for publication, and she agreed.

## 3. Discussion

In this case, we describe surgical resection and reconstruction of a second recurrence of giant cell tumor of the first metatarsal. Nonsurgical risk factors for recurrence are controversial, with some authors discounting all risk factors [12, 13]. Others endorse younger age; location in the distal radius, proximal tibia, and proximal femur; increasing Campanacci grade; larger soft-tissue mass; and bone envelope destruction to have higher rates of recurrence [14]. In addition, recurrence is related to the intervention chosen. Although nonoperative methods are being utilized more frequently, operative intervention is most commonly pursued and includes curettage with or without bone grafting, curettage with local adjuvants, en bloc resection, and amputation. Standard or first-line treatment of long bone GCT is intralesional curettage. A recent systematic review of GCTB in the small bones of hands and feet reported a 72% recurrence rate with curettage alone [6].

Local adjuvants are often added to decrease the recurrence rate and include phenol, liquid nitrogen (cryosurgery), hydrogen peroxide, ethanol, argon, high-speed burr, and polymethylmethacrylate. This patient was initially treated with curettage and PMMA cementation. Multiple studies suggest that this method is more effective in decreasing the chance of recurrence compared to curettage alone, with rates ranging from 14.3% to 22% [7, 8, 11]. This may be due to thermal necrosis of superficial tumor cells at the time of cementation [15].

Bone grafting is often employed to fill the resultant bony defect because there is no risk of disease transmission, there is no donor-site morbidity, and it provides sufficient mechanical support for early mobilization and rehabilitation. However, this method does not appear to drastically decrease the rate of recurrence. One study exhibited 45% recurrence in 677 patients treated with this method [16]. Since this patient had already undergone previous curettage, it was reasonable that the outside surgeon chose to use bone substitute graft in her second procedure, as it can fill defects and has osteoconductive qualities.

Multiple studies have also investigated systemic medical therapy designed to stabilize local and metastatic disease. Bisphosphonates inhibit GCTB-associated osteoclast resorption and promote osteoclast apoptosis. Some studies have demonstrated stabilization of disease with bisphosphonate-aided treatments, although there is heterogeneity in the regimens and specific medications utilized [17, 18]. Denosumab, a RANKL inhibitor that inhibits osteoclast maturation, is used to treat osteoporosis and skeletal pathology secondary to bony metastases. It is also approved by the U.S. Food and Drug Administration for use in unresectable GCTB. A phase two study of denosumab with patients with recurrent or unresectable GCTB demonstrated a favorable response in 86% of patients, and interim analysis of another study demonstrated 96% of patients without disease progression at 13-month follow-up [19, 20]. While these results are encouraging, further studies are needed to demonstrate the safety and long-term efficacy of systemic therapy.

Two years after the first recurrence, the patient presented to our institution with a second recurrence. En bloc excision is often used for recurrent or recalcitrant cases, such as the one presented in this report. However, there are conflicting views, and many authors advocate utilizing curettage with adjuvants even for multiply recurrent tumors to preserve the native joint [8, 12, 21]. In general, wide resection results in lower recurrence rates than local methods [2, 21–24]. However, when Oliveira et al. specifically investigated the small bones of hands and feet, the recurrence rate was higher following resection than curettage with adjuvants [6]. In addition, wide resection often results in reduced functional outcomes [6, 25–27]. Amputation can be performed but has both cosmetic and functional disadvantages.

In general, the chosen procedure should eradicate neoplastic tissue while preserving maximal function. Given that this patient had only a single cortex of her metatarsal remaining, the defect was uncontained and it was felt that the lesion had a very high risk of recurrence with repeat curettage, especially in the setting of a second recurrence with previous use of multiple local adjuvants. Greater morbidity was accepted in this case to prevent local recurrence and pulmonary metastasis, which has been shown to be increased in patients with recurrent disease [28]. In addition, the surgical choice was felt to be amenable to limb-sparing surgery given that the defect was reconstructible with a structural bone graft.

Excision usually requires reconstruction; methods include arthrodesis, extensive bone grafting, and bulk or structural allografts. Multiple case reports of GCT of the first metatarsal treated with excision and reconstruction with either iliac crest or fibular graft have been reported with no signs of recurrence and resumption of normal activities at short to midterm follow-up [29, 30]. Previous studies have also examined the efficacy of excision and reconstruction following the first recurrence of GCTB in the first metatarsal. Balaji et al. reported on two cases of recurrence in adolescents, one of which underwent Boyd's amputation. The second patient had a previous resection and unspecified reconstruction, and then underwent excision and ipsilateral fibular autograft, with return to regular activities with no signs of recurrence at 1-year follow-up [31]. Another study details the use of first metatarsal allograft for reconstruction following excision of recurrent GCT of the first metatarsal [32]. None, however, have examined treatment for multiply recurrent GCT of the first metatarsal as we have in this report. Iliac crest autograft was chosen in this case due to its established efficacy in foot and ankle arthrodesis surgery, a higher concentration of osteoprogenitor cells than grafts from the lower extremity, and relatively low donor-site pain [33–35].

In summary, nonoperative methods are being used more frequently to treat GCTB, including the use of systemic therapy such as denosumab. Operative treatment is most commonly pursued, with intralesional curettage as the appropriate first step. Local adjuvants are often added to decrease the recurrence rate, and multiple studies suggest its increased efficacy compared to curettage alone. En bloc excision and even amputation can be used for recurrent or recalcitrant cases, such as in this case. The first recurrence in small bones typically occurs before two years, although it is possible for multiple recurrences to occur years after the initial presentation [6]. This patient will require further surveillance, but at the time of this report she has achieved an excellent functional outcome with no signs of another recurrence.
